# Comparative evaluation of continuous renal replacement therapy and intermittent hemodialysis for the treatment of acute kidney injury

**DOI:** 10.1097/MD.0000000000045094

**Published:** 2025-10-31

**Authors:** Ping Li, Zhou Wu, Mingrui Zhao, Fen Chen

**Affiliations:** a Department of Kidney Disease, General Hospital of Central Theater Command, Wuhan City, Hubei Province, China.

**Keywords:** acute kidney injury, continuous renal replacement therapy, hemodynamic stability, intermittent hemodialysis, treatment outcome

## Abstract

Acute kidney injury (AKI) remains a critical clinical condition with high morbidity and mortality. Continuous renal replacement therapy (CRRT) and intermittent hemodialysis (IHD) are commonly employed renal support modalities. This study aimed to compare the therapeutic efficacy and clinical outcomes of CRRT and IHD in patients with AKI. A retrospective cohort study was conducted at a single tertiary care center from January 2022 to December 2024. A total of 256 patients diagnosed with AKI were included, comprising 136 patients treated with CRRT and 120 with IHD. Patients were selected based on Kidney Disease: Improving Global Outcomes criteria, excluding those with stage 4 to 5 chronic kidney disease (CKD), prior renal transplantation, significant comorbidities, or concurrent enrollment in other clinical trials. Key outcome measures included changes in hemodynamic parameters (mean arterial pressure and heart rate), renal function markers (serum creatinine and blood urea nitrogen), and Acute Physiology and Chronic Health Evaluation II scores. Statistical analyses were performed using SPSS version 27.0 (IBM Corporation, Armonk), with independent *t* tests and Chi-square tests applied where appropriate. Following treatment, mean arterial pressure decreased slightly in the CRRT group, whereas a statistically significant greater reduction was observed in the IHD group (*P* < .05). Heart rate increased in both groups; however, the elevation was significantly more pronounced in the IHD group. Both modalities resulted in improved renal function and a significant reduction in Acute Physiology and Chronic Health Evaluation II scores. The incidence of treatment-related complications was comparatively lower in the CRRT group. Survival analysis indicated that survivors were significantly younger and exhibited a lower prevalence of multi-organ dysfunction. Compared to IHD, CRRT was associated with improved hemodynamic tolerance, lower complication rates, and potentially better short-term outcomes in patients with AKI. These findings support the preferential use of CRRT in critically ill patients with hemodynamic instability and warrant validation through prospective, multicenter studies.

## 1. Introduction

Acute kidney injury (AKI), also known as acute renal failure, represents a critical medical condition characterized by an abrupt decline in renal function. The incidence of AKI has been escalating globally, attributed to various factors including increased exposure to nephrotoxic agents, advanced medical procedures, and a rising prevalence of chronic health conditions.^[1,2]^ This condition necessitates immediate intervention due to its potential to cause severe electrolyte imbalances, volume overload, and waste accumulation, leading to a high mortality rate.^[3]^

Continuous renal replacement therapy (CRRT) has emerged as a significant modality in managing AKI, particularly in hemodynamically unstable patients. CRRT is advantageous for its continuous nature, offering gradual solute and fluid removal over extended periods. This method is less likely to induce hemodynamic instability compared to intermittent strategies.^[4,5]^ The slow and steady modality of CRRT is crucial for patients with significant comorbidities, where rapid fluid shifts can be detrimental. Intermittent hemodialysis (IHD), the conventional approach in renal replacement therapy, involves periodic sessions typically lasting for several hours.^[6]^ IHD is effective in rapidly correcting fluid overload, electrolyte imbalances, and removing uremic toxins. However, its intermittent nature might cause fluctuations in hemodynamic status, particularly in patients with cardiovascular instability. Despite these concerns, IHD remains a widely used and effective treatment modality in many clinical settings.^[7]^

CRRT offers better hemodynamic stability and is preferable for patients with severe hemodynamic compromise. However, it requires sophisticated equipment and continuous anticoagulation, which can be challenging in certain clinical scenarios. On the other hand, IHD, with its ability to rapidly correct metabolic derangements, is more suitable for stable patients. Nevertheless, its intermittent nature can be a disadvantage for critically ill patients.^[8]^ This study aims to conduct a comparative analysis of CRRT and IHD in the management of AKI. By evaluating the therapeutic efficacy, patient outcomes, and potential complications associated with each modality, the study seeks to provide a comprehensive understanding of their roles in different clinical scenarios. This research is crucial for guiding clinicians in making informed decisions regarding the most appropriate renal replacement therapy for patients suffering from AKI. We hypothesized that, compared with intermittent hemodialysis, continuous renal replacement therapy would provide superior hemodynamic stability, result in fewer treatment-related complications, and be associated with improved short-term clinical outcomes in critically ill patients with AKI.

## 2. Materials and methods

### 2.1. Study design

A retrospective comparative study was conducted at our institution to evaluate the therapeutic efficacy of CRRT versus IHD in the management of AKI. The study period extended from January 2022 to December 2024. A total of 256 patients were included: 136 patients who received CRRT comprised the observation group, while 120 patients undergoing IHD formed the control group. Written informed consent was obtained from all participants. The study was reviewed and approved by the General hospital of central theater command Ethics Committee (Approval No. 2021-EC-056) in accordance with the Declaration of Helsinki.

### 2.2. Inclusion and exclusion criteria

Inclusion criteria:

1. A confirmed diagnosis of AKI based on Kidney Disease: Improving Global Outcomes criteria: an increase in serum creatinine by ≥0.3 mg/dL (≥26.5 μmol/L) within 48 hours, or an increase to ≥1.5 times the baseline value within the preceding 7 days.
2. Age ≥ 18 years, with no restriction on gender.
3. Receipt of either CRRT or IHD as the primary modality for AKI management during the study period.
4. Provision of written informed consent for study participation.

Exclusion criteria:

1. Preexisting stage 4 or 5 chronic kidney disease or patients undergoing maintenance dialysis prior to AKI diagnosis.
2. Presence of severe comorbid conditions not directly attributable to AKI, such as advanced malignancies or end-stage heart failure.
3. History of kidney transplantation within the preceding 6 months.
4. Concurrent participation in other clinical studies that could affect the current study outcomes.

### 2.3. Operational procedures for CRRT and IHD in AKI management

In the CRRT group, vascular access was obtained via catheterization of either the femoral or internal jugular vein. Continuous veno-venous hemofiltration was employed as the renal support modality. Anticoagulation was achieved using unfractionated heparin or low-molecular-weight heparin, unless contraindicated due to recent surgery or bleeding risk. The replacement fluid followed the standard ``Port formula’’ with individualized sodium correction using 10% sodium chloride to maintain a serum sodium gradient ≤ 15 mmol/L. Blood flow rates were maintained between 150 to 250 mL/min. Pre-dilution replacement fluid was administered at 2000 to 3000 mL/h, and post-dilution volumes were set at 600 mL/L. Ultrafiltration rates were adjusted according to urine output, hemodynamic status, and overall fluid balance. The duration of CRRT ranged from 3 to 5 days.

Patients in the IHD group underwent standard intermittent hemodialysis procedures. Dialysis sessions were prescribed based on the clinical status of the patient, with each session lasting approximately 5 hours. Anticoagulation was achieved with low-molecular-weight heparin, titrated according to individual risk profiles.

During the treatment period for both groups, continuous noninvasive monitoring of blood pressure and electrocardiography was implemented. Daily peripheral blood samples were collected in the early morning to assess serum electrolytes, liver and renal function, and arterial blood gas parameters.

### 2.4. Data collection and comparative analysis in CRRT and IHD treatment of AKI

Hemodynamic parameters, including mean arterial pressure (MAP) and heart rate (HR), were recorded before and after treatment. Fasting venous blood samples were analyzed to determine serum creatinine and blood urea nitrogen (BUN) levels. The Acute Physiology and Chronic Health Evaluation II (APACHE II) scoring system was applied to quantify illness severity based on acute physiological disturbances, age, and preexisting chronic health conditions. A 28-day follow-up period was used to record mortality outcomes in both groups.

### 2.5. Statistical analysis

All statistical analyses were performed using SPSS software version 27.0 (IBM Corp., Armonk). Data were classified as either quantitative or categorical. Normality of distribution was assessed using appropriate tests. For normally distributed continuous variables, comparisons between groups were performed using independent-samples *t* tests, and data were expressed as mean ± standard deviation. For non-normally distributed variables, data were expressed as median (interquartile range), and the Mann–Whitney *U* test was applied. Categorical variables were presented as frequencies and percentages, and comparisons were made using the Chi-square (*χ*²) test. In addition, multivariate logistic regression analysis was performed to adjust for baseline factors and potential confounders, including age, gender, baseline APACHE II score, presence of multi-organ dysfunction, baseline MAP, baseline HR, serum creatinine, and BUN levels, when assessing the association between treatment modality and clinical outcomes. A 2-sided *P*-value < .05 was considered statistically significant.

## 3. Results

### 3.1. Comparative analysis of hemodynamic parameter alterations in CRRT and IHD modalities

In the evaluation of hemodynamic stability post-renal replacement therapy, significant changes were observed within and between the CRRT and IHD groups. In the CRRT group, MAP decreased modestly following treatment, whereas the IHD group demonstrated a substantially greater decline. Additionally, both groups showed an increase in HR after treatment, with the IHD group experiencing a statistically significant greater rise compared to the CRRT group (Table 1).

**Table 1:** Inter-group comparison of mean arterial pressure (MAP) and heart rate (HR) before and after treatment.

Group	Number of patients	MAP before treatment (mm Hg)	MAP after treatment (mm Hg)	HR before treatment (beats/min)	HR after treatment (beats/min)
CRRT group	136	72.4 ± 7.4	70.7 ± 6.0*	116.5 ± 13.1	121.0 ± 9.4*
IHD group	120	71.3 ± 6.9	64.2 ± 5.0*^,^†	116.8 ± 13.7	132.2 ± 10.5*^,^†

CRRT = continuous renal replacement therapy, HR = heart rate, IHD = intermittent hemodialysis, MAP = mean arterial pressure.

* Indicates statistical significance within the same group (*P* < .05).

† Indicates statistical significance between groups posttreatment (*P* < .05).

### 3.2. Comparative analysis of Scr, BUN levels, and APACHE II scores pre- and posttreatment

An analysis of biochemical and clinical severity indicators revealed significant improvements posttreatment in both CRRT and IHD groups. Scr levels and BUN concentrations, key markers for renal function, showed notable reductions post-intervention. This trend was accompanied by a substantial decrease in the APACHE II scores, indicating an amelioration of clinical severity following treatment. The intergroup comparison posttreatment highlighted statistically significant differences, underscoring the distinct biochemical responses elicited by the 2 dialysis modalities (Table 2).

**Table 2:** Biochemical and clinical severity indicators in CRRT and IHD modalities.

Group	Scr before treatment (µmol/L)	Scr after treatment (µmol/L)	BUN before treatment (mg/L)	BUN after treatment (mg/L)	APACHE II score before treatment	APACHE II score after treatment
CRRT group	677.8 ± 67.5	186.4 ± 17.5*	36.8 ± 10.1	11.8 ± 3.7*	27.6 ± 6.5	13.7 ± 2.6*
IHD group	673.6 ± 68.0	257.6 ± 20.8*^,^†	37.9 ± 13.9	20.5 ± 7.5*^,^†	26.4 ± 7.4	17.4 ± 3.5*^,^†

BUN = blood urea nitrogen, CRRT = continuous renal replacement therapy, IHD = intermittent hemodialysis, Scr = serum creatinine.

* Indicates statistical significance within the same group (*P* < .05).

† Indicates statistical significance between groups posttreatment (*P* < .05).

### 3.3. Comparative analysis of complication rates in CRRT and IHD modalities

Upon analyzing the complication rates in patients undergoing CRRT and IHD, a distinct pattern emerged. The CRRT group experienced a lower overall incidence of complications (20.59%) compared to the IHD group (38.33%). A statistical examination utilizing the Chi-square test yielded a value of 4.06, with a *P*-value of .044, indicating a statistically significant difference between the 2 groups (Table 3).

**Table 3:** Incidence of complications in CRRT and IHD treatment groups.

Complication type	CRRT group (n = 136)	IHD group (n = 120)	*χ*²	*P*-value
Bleeding complications	4 cases (2.94%)	12 cases (10%)		
Catheter thrombosis	6 cases (4.41%)	10 cases (8.33%)		
Catheter site infections	8 cases (5.88%)	6 cases (5%)		
Catheter site hematomas	8 cases (5.88%)	8 cases (6.67%)		
Inadequate blood flow	2 case (1.47%)	10 cases (8.33%)		
Total complication incidence	28 (20.59%)	46 (38.33%)	4.06	<.05

CRRT = continuous renal replacement therapy, IHD = intermittent hemodialysis.

### 3.4. Prognostic indicators in AKI management

The univariate analysis of prognostic factors within the study population delineated clear distinctions between survivor and deceased groups. The mean age of survivors was significantly lower at 36.3 years, in contrast to 51.6 years in the deceased group. A striking 97.2% of the deceased group experienced multi-organ dysfunction, which was significantly higher compared to 34.8% in the survivor group. Admission APACHE II scores, indicative of disease severity at presentation, were considerably higher in the deceased group, averaging 25.9, versus 12.8 in the survivor group. All observed differences were statistically significant, with *P*-values <.05 (Table 4).

**Table 4:** Univariate analysis of predictive factors for prognosis.

Variable	Survivor group (n = 184)	Deceased group (n = 72)	*P*-value
Age	36.3 ± 10.5	51.6 ± 11.5	<.05
The occurrence of dysfunction in 2 or more organs	32 (34.8%)	56 (97.2%)	<.05
APACHE II score on admission	12.8 ± 5.1	25.9 ± 5.1	<.05

APACHE II = Acute Physiology and Chronic Health Evaluation II, ICU = intensive care unit.

## 4. Discussion

AKI remains a formidable challenge in critical care, with high incidence rates contributing to significant morbidity and mortality. Despite advances in clinical management, the outcomes for AKI are often contingent on early detection and the timely initiation of renal replacement therapy.^[9]^ CRRT and IHD have emerged as the principal modalities for managing severe cases of AKI, each with distinct physiological implications and logistical considerations.^[10]^ CRRT is often favored for hemodynamically unstable patients due to its gradual solute removal, while IHD is traditionally selected for hemodynamically stable patients given its efficiency in rapidly correcting fluid and electrolyte imbalances. This study contributes novel insights into the comparative effectiveness of CRRT and IHD, revealing the nuances in their impact on patient prognosis.^[11,12]^ By highlighting the relationship between treatment modality and critical outcomes such as multi-organ dysfunction and survival rates, our findings underscore the importance of personalized treatment strategies in AKI. In clinical practice, CRRT may be preferable not only for patients with hemodynamic instability but also for those with severe volume overload unresponsive to diuretics, cerebral edema or increased intracranial pressure, severe metabolic acidosis, or profound electrolyte imbalances refractory to medical management. Furthermore, CRRT allows precise fluid balance control in patients with multi-organ dysfunction, which can be critical in preventing further physiological deterioration.

The results suggest that while both CRRT and IHD are effective in managing the hemodynamic parameters of patients with AKI, there are discernible differences in their impact. The reduction in MAP may be attributable to intravascular volume removal and decreased vascular resistance, both recognized as physiological effects of dialysis. The more pronounced decline in MAP in the IHD group likely reflects the intermittent treatment schedule, which can predispose patients to more severe intradialytic hypotension.^[13]^ The posttreatment increase in HR may represent a compensatory mechanism to preserve cardiac output in response to alterations in blood pressure and intravascular volume status. The greater increase in HR in the IHD group corresponds with the larger MAP fluctuations observed, indicating a more pronounced cardiovascular response to intermittent hemodialysis.

The observed decrease in Scr and BUN levels posttreatment aligns with the primary objectives of renal replacement therapies, which are to mitigate the effects of renal failure by reducing the accumulation of renal toxins. The more pronounced reduction in Scr levels in the CRRT group suggests a more effective clearance in comparison to IHD, which may be attributable to the continuous nature of the therapy allowing for more stable toxin removal. The decrease in APACHE II scores across both treatment modalities posttreatment is indicative of an overall improvement in patient status, encompassing both physiological and chronic health variables.^[14]^ However, the IHD group, despite improvements, retained higher APACHE II scores compared to the CRRT group, suggesting that patients undergoing IHD may have a slower recovery trajectory or that CRRT may be more effective in managing critically ill patients.

The reduced complication rates in the CRRT group suggest a comparatively more favorable safety profile for this modality in the management of patients with AKI, potentially attributed to the continuous nature of the therapy. The types of complications varied, with bleeding complications and inadequate blood flow being notably higher in the IHD group. This finding may reflect the hemodynamic challenges and coagulopathies often associated with intermittent dialysis modalities. Catheter-related complications were present in both groups, underscoring the importance of meticulous catheter care in all renal replacement therapies.^[4]^ The clinical implications of these findings suggest that while both CRRT and IHD are indispensable treatments for AKI, careful consideration should be given to the patient’s condition when choosing the modality, to minimize potential complications.

The identified prognostic factors align with established clinical observations that older age and higher APACHE II scores are associated with poorer outcomes in acute medical conditions. The pronounced disparity in multi-organ dysfunction between the deceased and survivor groups underscores the critical impact of organ failure on survival prospects. The APACHE II scoring system’s effectiveness as a prognostic tool is reinforced by the substantial divergence in scores between the groups, correlating with patient survival.^[15]^ The significant association of these variables with mortality outcomes accentuates the necessity for early identification and intervention in at-risk populations. The management strategies for AKI must, therefore, prioritize the evaluation and stabilization of organ functions and address the heightened risks that older age groups face.

Despite the insights provided by this study, several limitations should be acknowledged. First, the retrospective design may introduce selection bias and precludes establishing causality. Second, the single-center setting may limit generalizability to other populations. Third, although the sample size was adequate for analysis, it may not capture the full variability of AKI presentations, especially rare complications. Fourth, while multivariate logistic regression was used to adjust for baseline factors, other confounding control methods such as propensity score matching were not applied. Fifth, potential confounders (including concurrent treatments, variations in dialysis protocols, timing of initiation, supportive care differences, as well as variations in operator experience, dialysis equipment, and institutional workflow) were not fully controlled, which may have influenced outcomes. Finally, the 28-day follow-up limited the evaluation of long-term renal recovery and survival. Future studies should include multicenter prospective designs, apply advanced confounding control techniques, extend follow-up, and incorporate patient-centered outcomes to better compare CRRT and IHD in AKI management.

## 5. Conclusions

In conclusion, CRRT appears to be preferable in selected hemodynamically unstable patients with severe AKI. It may help maintain hemodynamic stability, contribute to partial restoration of renal function, present a lower complication rate, and potentially reduce mortality. However, these findings should be interpreted with caution and warrant validation through further prospective research to confirm the benefits and optimize treatment protocols.

## Author contributions

**Conceptualization:** Ping Li, Zhou Wu, Fen Chen.

**Data curation:** Mingrui Zhao, Fen Chen.

**Formal analysis:** Mingrui Zhao, Fen Chen.

**Investigation:** Ping Li, Fen Chen.

**Methodology:** Ping Li, Fen Chen.

**Supervision:** Zhou Wu, Fen Chen.

**Validation:** Zhou Wu, Fen Chen.

**Visualization:** Fen Chen.

**Writing – original draft:** Ping Li, Fen Chen.

**Writing – review & editing:** Ping Li, Fen Chen.
